# Interaction of Microplastics, Plants, and Rhizosphere: A Critical Review

**DOI:** 10.3390/molecules31173028

**Published:** 2026-08-28

**Authors:** Ying Guo, Duo Zhang, Wenxin Li, Yuntao Zhao, Wei Su, Yi Xing, Chen Hong, Jianchao Wang, Yong Cui, Han Zhang, Jiayu Chen, Bo Jiang

**Affiliations:** 1 School of Energy and Environmental Engineering, University of Science & Technology Beijing, Beijing 100083, China; gy18531861867@163.com (Y.G.); zhangduoustb@163.com (D.Z.); lwx19861107891@163.com (W.L.); 18431256309@163.com (Y.Z.); suwei@ustb.edu.cn (W.S.); hongchen@ustb.edu.cn (C.H.); b2306890@ustb.edu.cn (H.Z.); 2 National Engineering Laboratory for Site Remediation Technologies, Beijing 100015, China; 3 Beijing Key Laboratory of Resource-Oriented Treatment of Industrial Pollutants, University of Science & Technology Beijing, Beijing 100083, China; 4 College of Environmental Science and Engineering, Hebei University of Science and Technology, Shijiazhuang 050018, China; xingyi@ustb.edu.cn; 5 State Key Laboratory of Nutrient Use and Management, National Academy of Agriculture Green Development, Key Laboratory of Plant-Soil Interactions (Ministry of Education), College of Resources and Environmental Sciences, China Agricultural University, Beijing 100193, China; jcwang91@cau.edu.cn; 6 School of Chemistry and Biology Engineering, University of Science and Technology Beijing, Beijing 100083, China; ycui9066@ustb.edu.cn; 7 China Research Institute for Science Popularization, Beijing 100081, China; jiayu1217@163.com

**Keywords:** microplastics, plants, soil, microbial communities, accumulation

## Abstract

Over the past decade, microplastic pollution has emerged as a subject of considerable interest and extensive research, with implications for human health and ecosystems. This paper briefly summarized the sources of microplastics and their distribution in the soil. It comprehensively addressed the effects of microplastics on the soil–plant system, including the impacts on soil physicochemical properties and plant rhizosphere microbial communities. The effects of microplastics on plant growth, along with their transformation and accumulation within plants, were evaluated. Microplastics can adhere to soil particles and root surfaces and, under certain conditions, may associate with outer root tissues or enter plants through damaged or vulnerable sites. Their presence in the soil–plant system may interfere with water and nutrient uptake, affect photosynthesis, and induce cytotoxic or genotoxic responses. Furthermore, the co-occurrence of microplastics with toxic substances or soil remediation materials may exacerbate the adverse effects on plants and ecosystems. Future research should focus on the development of methods for detecting microplastics in soils and plants and investigate the interactions between microplastics and other environmental factors within the soil–plant system. Further investigation is required regarding the role of microplastics in hyperaccumulating plants, particularly concerning plant-based methods for removing heavy metal pollutants. This study establishes a scientific basis for understanding the effects of microplastics on soil–plant systems.

## 1. Introduction

Synthetic polymer plastics are mass-produced and widely used because of their light weight, durability, versatility, and low cost [1]. They are among the top five most extensively manufactured materials worldwide [2]. Plastic pollution is a major global environmental problem. Plastics are stable and resistant to degradation but can fragment into particles smaller than 5 mm, known as microplastics, through physical and chemical processes, such as hydrolysis, photodegradation, biodegradation, and mechanical shear forces. Microplastics were first reported in the marine environment in 2004 [3]. Microplastics have attracted considerable attention because of their small size, ecological risks, and potential adverse effects on environmental and human health [4]. Microplastics are generally classified as primary or secondary microplastics, including commonly used materials such as polypropylene (PP), polyethylene (PE), polystyrene (PS), polyvinyl chloride (PVC), and polyester (PES) [5]. Microplastics can adsorb and release hazardous substances, such as pathogens, heavy metals, and organic contaminants [6], which can bioaccumulate and biomagnify through food webs, leading to environmental and organismal contamination [7,8].

Because of their ease of transportation and persistence in the environment [9,10], microplastics are frequently detected in water, atmosphere, soils, sediments, and Arctic regions [11]. Significant efforts have been dedicated to investigating the consequences of microplastics in aquatic environments, particularly marine systems, with studies showing that microplastics can alter the chemical properties and oxygen content of water. Research findings indicate that plastic is found in soil environments at concentrations 4–23 times higher than those in ocean environments [12]. Consequently, the soil environment is considered a significant source and sink for microplastic pollution [13]. Extensive research has demonstrated that microplastics can alter the physical and chemical properties of soils, thereby affecting the microbial community structure and elemental cycling processes [14,15]. Plants are fundamental components of terrestrial ecosystems, and research has examined the effects of microplastic exposure on plants. Microplastics can influence soil properties, enzyme activities, and rhizosphere microorganisms, leading to indirect effects on plant growth. Additionally, some studies have suggested that microplastics, alone or together with co-occurring pollutants, may be taken up by plants through root-associated entry pathways, thereby potentially affecting plant development and function [16]. In particular, microplastics can potentially affect the rhizosphere environment and gene expression in plants by interacting with root systems, leaf surfaces, and seeds [17,18]. However, compared to microplastics in marine ecosystems, their effects on soil ecosystems, particularly on plants, are relatively understudied.

It is important to review the published literature on the behavior of microplastics in soil ecosystems to gain a deeper understanding of their effects on soil–plant systems. To provide a general overview of the development of this research field, a brief bibliometric overview was conducted based on the Web of Science Core Collection. A Topic search was carried out using the terms “microplastics”, “soil”, and “plants”, and the retrieved records were analyzed using the built-in analytical functions of the Web of Science platform. According to the updated search results, 5195 records were identified. The annual number of publications increased substantially in recent years, reflecting the rapidly growing interest in this topic. The countries with the highest publication output were China, India, and Germany. In terms of research areas, the publications were predominantly classified under Environmental Sciences/Ecology, Public, Environmental & Occupational Health, and Toxicology, followed by Agriculture and Plant Sciences. Overall, these results provide a general picture of the publication trends and disciplinary distribution of research on microplastics in soil–plant systems.

Therefore, this study aimed to investigate the effects of microplastics on soil–plant systems, focusing on (1) the sources and distribution of soil microplastics in the soil; (2) the effects of microplastics on soil physicochemical properties, microbial communities, and plant growth; (3) the combined effect of microplastics and other pollutants or soil remediation materials on plants; and (4) the uptake, transport, and accumulation of microplastics by plants. This study also anticipates future research directions that are likely to include detection, degradation, and comprehensive risk assessment and management strategies for microplastics. This will facilitate a more accurate evaluation of the potential risks that microplastics may pose to ecosystems and human health, while also enabling the investigation of methods for the removal of microplastics from soil.

## 2. Sources and Distribution of Soil Microplastics

**Figure 1:**
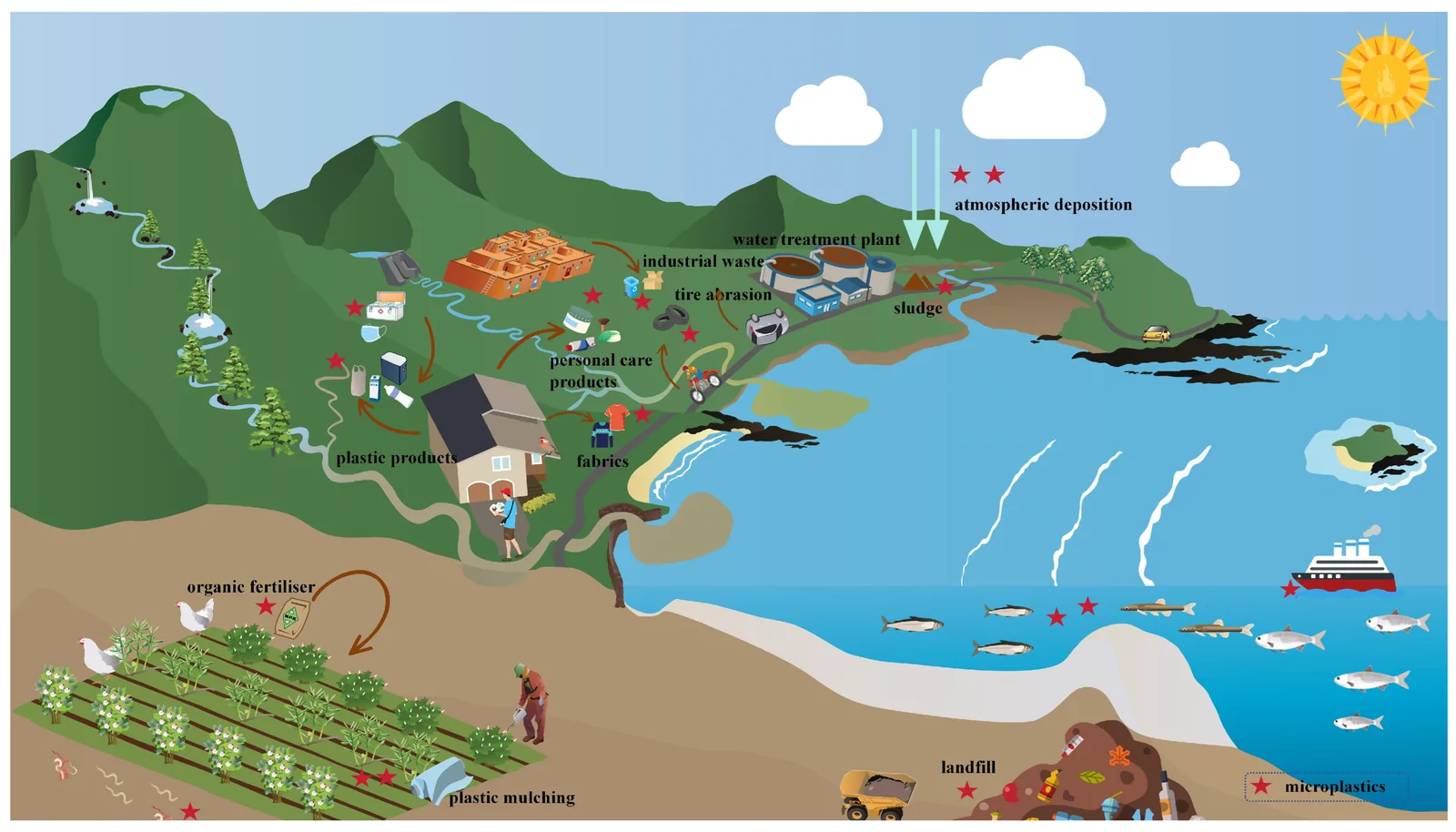
Several major sources of microplastics in soil.

Microplastics have been widely detected in soil environments, with reported abundances varying considerably across regions and land-use types. The sources of soil microplastics include the degradation of agricultural plastic mulching films [19], sewage sludge [20], and compost from agricultural activities [21,22]; improper disposal of plastic waste (e.g., food packaging, plastic bottles, and tea bags); release of textile-derived fibers [23]; use of cosmetics and personal care products [24]; and tire wear generated by road traffic [25]. Improper disposal of COVID-19-related plastic products, including disposable masks, gloves, and laboratory waste, contributes to the accumulation of microplastics in soils [26]. Atmospheric deposition is another significant source of microplastics in soil (Figure 1) [27].

The distribution of microplastics in soils is influenced by various factors, including the physical and chemical properties of microplastics, locations of pollution sources, and natural transport processes such as water movement and wind. Representative soil-based studies on the sources and distribution characteristics of microplastics are summarized in Supplementary Table S1. Anthropogenic factors, including industrial and agricultural activities, contribute to this distribution. Consequently, the distribution of microplastics in the soil environment is highly heterogeneous and complex. An in-depth study of the distribution characteristics and various influencing factors of microplastics in terrestrial environments, along with enhanced research on their detection, migration, degradation, and ecological effects, will be beneficial for reducing potential threats to ecosystems and public health, as well as for a better understanding of the extent of microplastic contamination.

## 3. Effects of Microplastics on Plants

Microplastics in soil can affect plants through both direct and indirect pathways. In addition to direct interactions with seeds, roots, or other plant tissues, microplastics may also modify soil physicochemical properties, nutrient dynamics, enzyme activity, and rhizosphere microbial communities, thereby influencing plant growth and development [28]. Because these processes are closely interconnected, microplastic-induced changes in the soil environment can propagate through the soil–plant-microbe continuum and ultimately alter plant performance. The magnitude and direction of these effects, however, are often context dependent and influenced by microplastic characteristics and environmental conditions. Findings from hydroponic or other simplified laboratory systems cannot be directly extrapolated to agricultural soils, where soil-specific processes can substantially alter microplastic behavior and bioavailability.

### 3.1. Effects of Microplastics on Soil Properties and Rhizosphere Microbial Communities

The rhizosphere represents a critical interface between plant roots and the surrounding soil environment. In soil–plant systems, microplastics can influence plant growth not only through direct interactions with roots, but also indirectly by altering soil physicochemical properties, nutrient availability, and rhizosphere microbial communities [29,30]. These changes may subsequently affect root functioning, nutrient acquisition, and overall plant performance. However, the impacts of microplastics on rhizosphere processes are often variable, reflecting differences in polymer characteristics, exposure levels, soil conditions, and experimental settings. In particular, soil-based responses are shaped by particle aggregation, aging, sorption to mineral and organic components, and interactions with rhizosphere microorganisms, all of which may alter the mobility and bioavailability of microplastics relative to hydroponic systems.

#### 3.1.1. Effects of Microplastics on Soil Physical, Chemical, and Nutrient Properties

Microplastics can alter soil physical structure and thereby affect water retention, evaporation, aeration, and root-zone water dynamics. As hydrophobic materials, microplastics may affect soil aggregates upon entering the soil environment [31], which is often associated with reduced soil water retention. Moreover, microplastics can affect the distribution and uptake of water in soil. Wan et al. found that increasing microplastic concentrations decreased soil structural integrity, resulting in greater soil water evaporation [32]. Similarly, PE has been shown to adversely affect soil water content, with the reduction being proportional to increasing microplastic concentration, and this was linked to decreased barley (*Hordeum vulgare*) yield [33]. During maize cultivation, microplastics were also reported to affect water interactions in the root zone, reducing water uptake by maize roots and adversely affecting plant growth [34]. In addition, because of their small size, microplastics may create barrier-like or discontinuous zones within the soil matrix, facilitating water vapour movement and enhancing evaporation; these effects appear to vary with particle size and concentration [35]. Microplastic particles may also reduce soil bulk density, thereby reducing resistance to root penetration and enhancing soil aeration. Although increased porosity and permeability may, in some cases, facilitate root growth, they can also negatively impact soil water retention. Taken together, these findings suggest that microplastics can substantially affect soil water relations; however, the magnitude and direction of these effects vary, and depend on polymer type, particle characteristics, concentration, and soil conditions.

Microplastics can also modify soil chemical properties, particularly pH and electrical conductivity (EC), both of which are closely linked to nutrient availability and root activity. For instance, the application of PP at a density of 1.17 g/cm^3^ to maize (*Zea mays* L.) soil resulted in significant alterations in soil pH and a remarkable increase in EC [36]. By contrast, exposure of wheat soil to low-density PE (LDPE) results in increased pH and decreased EC [35]. These contrasting responses indicate that the effects of microplastics on soil chemical conditions are highly context dependent. Several factors may contribute to changes in soil pH and EC, including the type and shape of microplastics, the presence of additives, and changes in enzyme activities [37]. Additionally, the extensive active surface area of microplastics may affect the ion exchange process in soils [38] because these anions and cations are closely linked to soil pH and EC. The effect of microplastics on soil nitrogen fixation and ammonification may further affect pH and EC by consuming more H^+^, thereby affecting soil conditions [39]. Alterations in soil pH and EC can indirectly affect plant growth. Given that plant roots are sensitive to pH, an appropriate pH range is essential for normal metabolic activity and shifts in pH may alter the form and availability of nutrients. These alterations can either inhibit or enhance nutrient activity, thereby affecting plant growth. Additionally, variations in soil EC may affect the nutrient uptake and balance in plants, resulting in nutrient imbalances and restricted growth. However, existing evidence does not support a single consistent pattern, highlighting the need to interpret pH and EC changes in relation to specific soil-microplastic combinations.

Microplastics also have the potential to affect soil nutrient cycling, particularly the cycling and availability of phosphorus (P), nitrogen (N), and carbon (C), thereby indirectly influencing plant growth. They may modify not only the content of available nutrients but also their forms and turnover, with possible consequences for rhizosphere processes and plant nutrient acquisition. For example, the incorporation of PE and polyacrylonitrile into soils following wheat cultivation led to a substantial decrease in ammonia (NH_3_) volatilization, ranging from 66.7% to 67.4%, while plant N uptake increased by 31.9% to 74.3% [40]. Sun et al. observed reduced concentrations of NH^4+^-N and NO_3_^−^-N in rice soils after the addition of various concentrations of PVC [41]. Furthermore, Sravya et al. found that LDPE treatment of green gram (*Vigna radiata* L. Wilczek) negatively impacted the plant’s capacity for nutrient uptake from the soil, resulting in decreased uptake of N, P, and K as the microplastic concentration increased [42]. The effects of microplastics on the availability of essential nutrients are contingent on their type, particle size, and concentration [43]. Since nutrient availability is fundamental to photosynthesis, energy metabolism, and root development, such changes may ultimately modulate plant growth. However, the reported responses are inconsistent across studies, suggesting that nutrient-related effects are also shaped by soil characteristics, crop species, and exposure duration. Overall, current evidence indicates that microplastics can alter soil nutrient dynamics; however, the underlying mechanisms remain poorly understood and require further verification under long-term, realistic soil conditions.

#### 3.1.2. Effects of Microplastics on Rhizosphere Microbial Communities

Microbial communities play a critical role in ecosystem functions. The structure and abundance of these communities are intricately linked to soil quality and fertility [12]. Microbial communities in the plant rhizosphere can decompose soil organic matter, remove toxins, facilitate nutrient cycling, stimulate plant growth, and prevent soil-borne infections [44,45]. Because of their small size, high specific surface area, and capacity to sorb organic compounds, microplastics may influence rhizosphere microorganisms not only by providing colonizable surfaces and potential carbon substrates, but also by altering the surrounding soil microenvironment [46]. Many studies have therefore examined the effects of microplastics on rhizosphere microbial communities, with reported responses varying according to polymer type, particle size, shape, concentration, aging state, and soil properties [47,48,49]. More importantly, these microbial responses should be interpreted within a broader framework in which microplastics modify soil physical structure, water availability, nutrient dynamics, and chemical conditions, which in turn shape rhizosphere microbial assembly and function.

Existing studies indicate that microplastics can alter rhizosphere community composition; however, these compositional changes are neither uniform nor necessarily indicative of equivalent functional outcomes. For instance, studies investigating soybean plants found that PBAT and PLA primarily increased the abundance of copiotrophic bacteria (*Proteobacteria*), while inhibiting oligotrophic bacteria (*Verrucomicrobiota*, *Gemmatimonadota*, etc.). Conversely, LDPE was found to enhance the abundance of oligotrophic bacteria (*Verrucomicrobiota*, etc.) [50]. Similarly, Lian et al. studied the effects of PE and PLA on the microbial community of soybean soil and found that 0.1% PE significantly increased the relative abundance of *Kocuria*, a member of the *Actinomycetes* phylum. The application of 0.1% PLA resulted in notable alterations in the abundances of several bacterial genera [51]. In potato-planted soils treated with 0.1%, 0.5%, and 5.0% LDPE, the relative abundances of *Bradyrhizobium* and *Devosia* increased, whereas that of *Phycicoccus* decreased [52]. Furthermore, PS, PE, and PP at concentrations of 0.01% and 0.1% were associated with a significant increase in *Haliangium* in the rhizosphere community [53]. However, such observations must be interpreted with caution. Most of these studies rely on relative abundance data derived from amplicon sequencing; shifts in relative abundance do not necessarily imply corresponding increases or decreases in absolute population size [54,55]. Moreover, since microbiome datasets are compositional, changes in one taxon can mathematically alter the apparent abundance of others; accordingly, conclusions based solely on relative abundance may overstate ecological significance in the absence of absolute quantification or appropriate compositional analyses [56,57].

Beyond taxonomy, a more informative question is whether microplastic-induced microbial shifts translate into functional consequences for soil and plant performance. The rhizosphere is critical for sustaining microbial diversity and mediating processes related to soil aggregation, organic matter turnover, nutrient transformation, and disease suppression. Specific microorganisms produce mucilage and extracellular polymeric substances that promote aggregate formation, thereby improving water retention and aeration [58]. Others contribute directly to nutrient acquisition, for instance via nitrogen fixation, phosphorus solubilization, and the mineralization of organic substrates into plant available forms. Beneficial rhizosphere microorganisms may also suppress pathogens; for instance, siderophore-producing *Pseudomonas* spp. can inhibit *Gaeumannomyces graminis* var. *tritici* and *Fusarium* spp. [59]. Therefore, changes in rhizosphere microbial composition are ecologically meaningful only when linked to functional indicators, including enzyme activities, nutrient transformation rates, functional genes, pathogen pressure, or plant performance. Accordingly, several recent studies and reviews have emphasized that microplastic-induced effects on microbial communities should be integrated with measurements of carbon and nitrogen cycling, soil enzyme activities, and plant outcomes, rather than being interpreted solely on the basis of taxonomic shifts [60,61,62].

The mechanisms underlying these microbial responses are likely multifaceted and context dependent. Microplastics may indirectly alter rhizosphere microbial communities by modifying soil physical structure, water retention, pH, redox conditions, and nutrient availability, all of which represent key drivers of microbial assembly and activity [63,64]. They may also exert more direct effects via additive release, adsorption and transfer of co-contaminants, or, for certain biodegradable polymers, supply of labile carbon that favours specific microbial taxa [65]. In addition, particle aging and weathering can modify surface chemistry, surface roughness, and the release of dissolved organic compounds, thereby further modulating microbial responses [66]. Plant-related factors, including crop species, developmental stage, and root exudation profiles, alongside soil texture, organic matter content, and baseline fertility, may further modulate these effects [67]. Taken together, these considerations suggest that microplastic impacts on rhizosphere microorganisms cannot be generalized across polymer types. In particular, biodegradable microplastics may differ from conventional microplastics with respect to both their degradation behavior and their interactions with soil microbial processes. Their effects on soil properties and rhizosphere microbial communities are therefore discussed separately in the subsequent section.

#### 3.1.3. Effects of Biodegradable Microplastics on Soil Properties and Rhizosphere Microbial Communities

Most existing studies investigating microplastics in soil–plant systems have focused largely on conventional polymers, whereas the effects of biodegradable microplastics remain relatively underexplored. This distinction is important, as biodegradable microplastics should not be assumed to act as functionally equivalent substitutes for conventional plastics within soil. Owing to their divergent chemical compositions, degradation pathways, and potential release of labile carbon and additives, biodegradable microplastics may exert distinct impacts on soil physicochemical properties and rhizosphere microbial communities. Available evidence suggests that biodegradable microplastics can alter soil conditions, although the direction and magnitude of these effects vary across studies.

For example, Inubushi et al. reported that the addition of biodegradable textile-derived microplastics to maize cropped soils decreased soil pH while increasing EC, indicating that biodegradable plastic inputs may modify the soil chemical environment [68]. In addition, the accumulation of mulch decomposition residues in cotton fields has been associated with soil quality deterioration and a marked decline in soil organic matter [69], suggesting that the long-term persistence and transformation of biodegradable plastic residues may exert unintended consequences for soil functioning. However, such findings should be interpreted with caution, since the observed responses may depend not only on polymer composition and degradation stage, but also on residue load, field management, and initial soil properties. Biodegradable microplastics may also alter rhizosphere microbial communities. However, current evidence remains limited and does not yet support broad generalizations. Liu et al. found that biodegradable microplastics reduced fungal α-diversity and network complexity in maize cropped soils, implying potential disturbances in fungal community stability and intertaxon interactions [70]. In wheat planted soils, biodegradable microplastics were reported to exert a stronger influence on rhizosphere bacterial communities, increasing the relative abundance of *Bacillus* and *Variovorax* [35]. Although such shifts may suggest selective stimulation of microorganisms capable of utilizing degradation products or tolerating altered soil conditions, taxonomic changes alone are insufficient to determine whether these responses are beneficial or detrimental to soil functioning and plant performance. Relative abundance data do not necessarily reflect changes in absolute abundance; accordingly, microbial compositional shifts should ideally be interpreted alongside measurements of enzyme activities, nutrient transformations, functional genes, and plant responses.

Mechanistically, the effects of biodegradable microplastics may differ from those of conventional plastics because their degradation can supply bioavailable carbon, alter local pH and salinity, and generate intermediate by-products that selectively modulate microbial growth. Meanwhile, additives, incomplete degradation, and the accumulation of residual fragments may also impose stress on soil biota or alter nutrient cycling [70,71,72]. Consequently, biodegradable microplastics may simultaneously act as carbon sources, physicochemical perturbants, and carriers of associated compounds, with the net outcome governed by polymer type, degradation status, exposure duration, soil properties, and crop species. Overall, current evidence suggests that biodegradable microplastics are not inherently benign in soil–plant systems, although their long-term effects on soil functioning and plant performance remain insufficiently understood.

### 3.2. Effects of Microplastics on Plant Growth and Development

The effects of microplastics on the growth, physiological functions, photosynthesis, and metabolites of plant seedlings have been systematically investigated. Considering that microplastics readily combine with other pollutants to form compound pollutants in the soil and that studies examining the effects of compound pollutants on plants remain limited, particular emphasis has been placed on investigating the effects of microplastics in conjunction with other soil pollutants or soil remediation materials. Overall, the available literature indicates that plant responses to microplastics are highly variable. While inhibitory effects are most commonly reported, stimulatory responses can also be observed under specific conditions. Such discrepancies do not necessarily represent contradictions; rather, they suggest that plant responses are context dependent and shaped by differences in polymer type, particle size and shape, concentration, exposure duration, crop species, developmental stage, and soil properties. These responses are also influenced by the exposure system, as observations from hydroponic or other simplified media may not accurately reflect particle behavior, exposure pathways, or bioavailability under heterogeneous soil conditions.

#### 3.2.1. Effects of Microplastics on the Growth of Plant Seedlings

The effects of microplastics on plant seedling growth can be assessed through seed germination and root growth, which are established bioindicators of plant toxicity following acute exposure [73]. Current studies broadly reveal two response patterns: (1) inhibition of germination and early growth, particularly at relatively high concentrations, and (2) neutral or even stimulatory effects under certain low-dose or species-specific conditions.

Growth inhibition has been documented in several crop species. For example, a study found that the incorporation of PS at a concentration of 50 mg/kg led to a reduction in the stem height and root weight of wheat seedlings, thus impacting their normal growth patterns [74]. Furthermore, PP, PVC and PLA at 0%, 0.05%, 0.1%, and 0.5% *w*/*w* inhibited the germination rate of coriander (*Coriandrum sativum* L.), resulting in 60–100% inhibition on day 7, and consequently affected the length of seedlings [75]. Biomass is a fundamental biological and functional characteristic of plants that can be influenced by microplastics. Urbina et al. demonstrated that the biomass of maize treated with PE was reduced by approximately 50%. The study further revealed that maize treated with high concentrations of PE exhibited a decreased total leaf area and stem/root ratio [76]. Similarly, Wang et al. observed that 10% PLA significantly reduced maize biomass [77].

In contrast, several studies have reported apparent stimulatory growth responses, particularly with respect to root traits. Treatments with PE and PS had a 97.5% probability of significantly increasing the root biomass of vegetative *Allium fistulosum* [78]. This suggests that microplastics do not exert universally negative impacts on seedling growth, and that responses may vary across species and plant organs.

Taken together, these studies indicate that seedling responses to microplastics often follow a non-linear pattern rather than a simple monotonic decline. One plausible explanation is that low levels of microplastics may transiently alter soil porosity, aeration, or water distribution in ways that favor root exploration, whereas higher concentrations may create mechanical barriers, reduce seed–soil contact, disrupt water uptake, or impair nutrient acquisition [79,80]. In addition, elevated root biomass should not automatically be interpreted as an overall beneficial outcome, since it may also reflect stress-induced biomass reallocation or compensatory growth [81]. Therefore, the divergent findings reported in the literature are likely driven by direct phytotoxicity, indirect soil-mediated impacts, and variations in experimental design.

#### 3.2.2. Effects of Microplastics on Plant Photosynthesis

Microplastics can affect plant metabolism and physiological processes, disturb hormone balance, and alter photosynthesis. Because plant growth and development are closely linked to photosynthetic performance, chlorophyll content is frequently used as an indicator of microplastic-induced stress. Most studies report reduced chlorophyll content and photosynthetic capacity, although stimulatory effects can also be observed under low-exposure conditions.

One group of studies documents inhibitory impacts on photosynthetic pigments and associated physiological performance. Wu et al. observed a reduction in chlorophyll content in *Oryza sativa* due to stress from PS, which led to a reduction in photosynthetic capacity [82]. Yang et al. reported that HDPE and general-purpose PS (GPPS) treatments reduce starch and chlorophyll content in the leaves of *Brassica chinensis* L. [83]. For tomato plants, 10% PVC to tomato plants resulted in a significant reduction in Chlorophyll a content by 24%. In addition, Chlorophyll b content was also found to be significantly reduced by 20% and 21% at 7.5% and 10% PVC, respectively. These results indicated that plant photosynthesis was compromised, which in turn inhibited growth and development [84]. In rice, increasing PLA concentrations significantly reduced photosynthetic activity, and the highest PLA concentration (2.5%) significantly decreased transpiration rate and stomatal conductance [85].

A second group of studies suggests that low microplastic doses may sometimes enhance photosynthetic traits. For example, the chlorophyll content of *Triticum aestivum* significantly increased under treatment with 0.1 mg/L and 1.0 mg/L of PS, which enhanced photosynthesis and promoted substantial seedling development [86]. Similarly, Liu et al. found that low concentrations of PE increase the levels of photosynthetic pigments in wheat plants, thereby enhancing photosynthesis and fostering plant growth [87].

Water-use efficiency, transpiration rate, and stomatal conductance represent key indicators of photosynthetic efficiency. Since soil constitutes a porous medium, microplastics can alter soil structure and biological activity, thereby indirectly modulating plant photosynthesis. These indirect effects may partly explain the variability in photosynthetic responses reported across studies.

Overall, the impacts of microplastics on photosynthesis appear to be concentration dependent. Low concentrations may occasionally promote photosynthetic performance by altering soil aeration or water distribution, whereas higher concentrations more frequently suppress photosynthesis via reduced chlorophyll synthesis, disturbed stomatal regulation, nutrient limitation, oxidative stress, and additive release. These responses are further governed by particle size, polymer type, aging status, exposure duration, and soil conditions.

#### 3.2.3. Effects of Microplastics on Antioxidant Enzyme Activities in Plants

Microplastics can enter and accumulate in plants, causing oxidative stress and affecting antioxidant enzyme activities, which may ultimately inhibit plant growth and development.

It is generally accepted that microplastics affect plant cells through both physical and chemical pathways, thereby elevating the production of reactive oxygen species (ROS). Although ROS function as important signaling molecules in plant stress responses, excessive ROS accumulation can damage cell membranes, proteins, RNA, and DNA, resulting in oxidative injury. Therefore, alterations in ROS levels and antioxidant-enzyme activities are commonly employed as indicators of microplastic toxicity in plants [88]. Numerous studies have indicated that microplastics can induce the overproduction of ROS in plants, potentially leading to oxidative damage and toxic effects [89,90]. For example, exposure of wheat plants to the plasticizers di-*n*-butyl phthalate (DBP) and di(2-ethylhexyl) phthalate (DEHP) increased ROS levels and reduced chlorophyll content and CO_2_ assimilation rate [91]. Additionally, long-term PE exposure led to ROS damage in lettuce (*Lactuca sativa* L.) leaves, with a significant increase in ROS content [92]. Also, Gao et al. applied a range of PE concentrations to lettuce (*Lactuca sativa* L), which showed that an increase in ROS concentration was more pronounced in plant roots in comparison to leaves and shoots, indicating that microplastics induce greater oxidative damage to plant roots than to leaves [93]. These findings support the notion that oxidative stress represents a key pathway via which microplastics impair plant physiological performance.

Superoxide dismutase (SOD), peroxidase (POD), and catalase (CAT) are key components of the antioxidant defense system [88]. SOD serves as the first line of defence against oxygen radicals, CAT scavenges H_2_O_2_ generated during metabolism, and POD also contributes to ROS detoxification [87]. Available studies reveal two main response patterns. Under moderate-stress conditions, antioxidant-enzyme activities may rise as part of a defensive response. For example, PS exposure significantly increased SOD and POD activities in broad bean (*Vicia faba* L.) root tips [94], and PP treatment elevated SOD and CAT activities in peanut plants [95]. In contrast, under stronger or prolonged stress, enzyme activities may be inhibited. Wu et al. found that SOD, POD, and CAT activities in rice (*Oryza sativa* L.) leaves were suppressed under PS exposure (250 and 500 mg/L) [82]. Similarly, 200 mg/L of PS markedly inhibited CAT activity in wheat [96], and high PE concentrations in wheat seedlings increased oxidative stress while significantly decreasing SOD and CAT activities [87]. These contrasting responses suggest that antioxidant enzymes may initially be activated as an adaptive response; however, they can be suppressed when stress exceeds the detoxification capacity of the plant.

#### 3.2.4. Effects of Microplastics on Plants at the Molecular Level

The effects of microplastics on plants at the molecular level have been increasingly investigated with the development of omics technologies. For instance, after exposure of broad bean (*Vicia faba* L.) to PS, the cells exhibited enlarged chromosome structures and increased DNA content. In addition, the mitotic index of the root tip cells was significantly reduced. Oxidative stress or damage to the antioxidant system results in DNA damage including strand breaks, base modifications, and DNA adduct formation, thereby causing genomic instability. This is regarded as the primary cause of genotoxicity [97]. For instance, after exposure of broad bean (*Vicia faba* L.) to PS, the cells exhibited enlarged chromosome structures and increased DNA content. In addition, the mitotic index of the root tip cells was significantly reduced [94]. The effects of microplastics on plant gene expression have direct effects, including abnormal gene expression and genotoxicity, and indirect effects, involving modifications to the soil environment and alterations in soil microbial communities. Physical damage caused by microplastics may trigger stress responses in plants, leading to the activation of genes associated with oxidative stress and their respective metabolic pathways and biological processes, which can be identified using transcriptomic techniques.

Investigating the interactions between microplastics and antioxidant systems through transcriptomics is essential for elucidating the resistance mechanisms of plants against microplastic stress. Li et al. exposed melon (*Cucumis melo* L.) seeds to PVC for transcriptome analysis, and the results demonstrated that a significant number of hormone-related genes, including the growth hormone gene, exhibited differential expression, affecting ROS levels, various metabolic pathways, and plant growth [98]. Furthermore, in lettuce treated with 0.2 μm PS at varying concentrations, transcriptome analysis identified six CAT-related genes and four ascorbate peroxidase-related genes, showing notable differences by PS concentration [99]. This indicates that the microplastic concentration significantly affects the transcript levels of antioxidant enzyme-related genes. However, Binjawhar et al. found that rice (*Oryza sativa*) seedlings exposed to PS, PVC and PE exhibited enhanced expression of genes related to antioxidant enzymes, resulting in an increase in plant resistance to microplastic toxicity [100]. It has been demonstrated that this process has a facilitating effect on plant growth and development. Similarly, the differential expression of 2983 and 1640 genes related to photosynthesis and energy metabolism was observed in buckwheat leaves exposed to 20 and 80 mg/L of PE, respectively [101]. Zhuang et al. investigated the effects of 5 and 0.1 μm of PS on photosynthesis in cucumber leaves at the molecular level [102]. Their findings suggested that low-concentration treatments may alleviate the adverse effects of microplastic exposure on photosynthetic efficiency by modulating the expression of specific genes. However, alterations in the expression of certain genes due to high-concentration treatments lead to a reduction in photosynthesis. The expression of genes involved in various metabolic processes in plants is closely connected to plant development, making it crucial to investigate their expression in response to microplastics. Overall, these responses appear to be highly dependent on microplastic type, concentration, and plant species.

Microplastics that penetrate and accumulate in plants can adversely affect plant growth by altering the expression of genes associated with specific metabolic pathways and biological processes. In rice plants subjected to PBAT and PE mulching, the genes encoding ammonium and nitrate nitrogen transporter proteins in the root system were downregulated at both stages of nutrient development. The phenylpropanoid biosynthesis pathway was inhibited, resulting in a reduction in lignin content, which adversely affected nitrogen and phenylpropane metabolism [103]. Similarly, biological pathway analyses revealed that PE stress disrupted several metabolic pathways in *Pisum sativum* seedlings. For example, the metabolic pathways of alanine, aspartic acid, and glutamic acid were significantly affected, which was adverse to plant growth [104]. Furthermore, Liu et al. examined cucumber plants treated with 5 and 0.1 µm PS, demonstrating that the synthesis of lignin and the expression of related genes was inhibited, indicating that PS treatment affected phenylpropane metabolism in the plants [105].

Research has indicated that the properties of microplastics can significantly affect plant metabolomics, leading to notable alterations in sugars and organic acids within plants. The variety of sugars in plants is crucial for energy storage and supply [106]. For instance, cotton exposed to PS exhibited an increase in monosaccharide abundance, indicating that sucrose hydrolysis may be activated to meet the energy requirements necessary to alleviate microplastic-induced stress in plants [107]. Wu et al. demonstrated that exposure of *Oryza sativa* L. to PS resulted in a reduction in the content of 16 sugars, 26 organic acids, and 17 additional metabolites [82].

**Figure 2:**
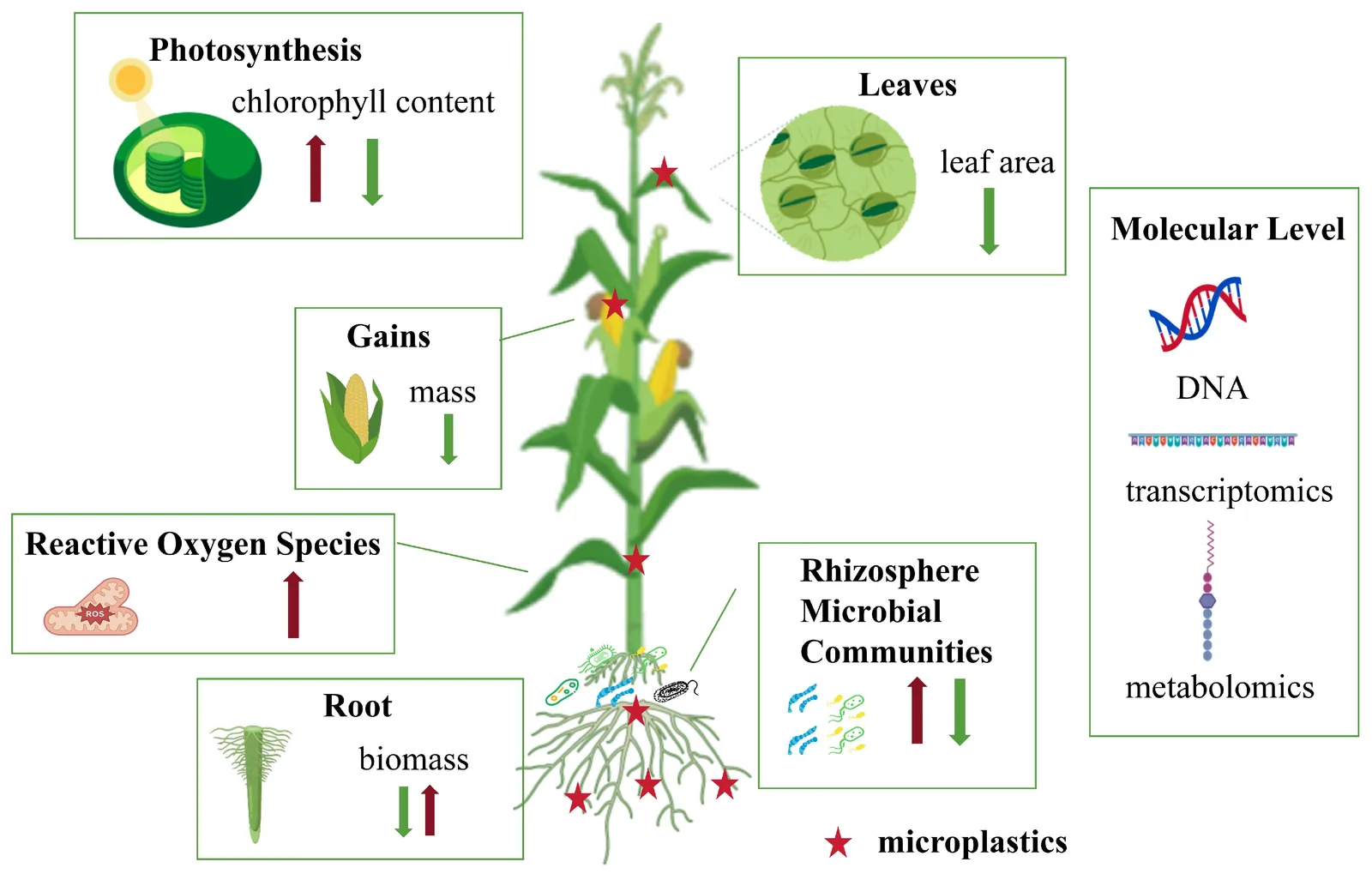
Plant growth and development responses to microplastic exposure. Red upward arrows indicate the increase of corresponding physiological indicators, while green downward arrows indicate the decrease of corresponding physiological indicators.

Plant cells use organic acids as intermediates in primary carbon metabolism. These acids serve as critical mediators of substance exchange between plants and their surrounding environment and play a significant role in nutrient activation and uptake, thereby aiding plants in coping with stress [108]. Shi et al. observed that tomato plants (*Lycopersicon esculentum* L.) subjected to microplastic stress exhibited a substantial increase in the concentration of organic acids present in their root exudates [109]. Furthermore, the application of PS to oilseed rape resulted in a marked elevation of citric acid and oxalic acid concentrations within root exudates [110]. Taken together, these findings suggest that microplastics can alter carbon metabolism and root exudation patterns, although the underlying molecular mechanisms remain to be fully elucidated (Figure 2).

Given the substantial variability in microplastic types, particle sizes, exposure durations, and environmental conditions, as well as inconsistent magnitudes of their effects on plants, further comprehensive research is urgently needed [15]. Future studies should more systematically compare evidence across exposure systems and relate observed plant responses to polymer type, particle size and shape, aging state, concentration, plant species, exposure duration, and the level of analytical confirmation. Particular attention should be paid to environmentally realistic concentrations and to long-term soil-based and field-relevant conditions, since these are essential for accurately assessing the ecological significance of microplastic impacts in plant–soil systems.

### 3.3. Combined Effects of Microplastics and Other Pollutants on Plants

The unique surface structures of microplastics, characterized by large specific surface areas, cracks, and pores, enable them to adsorb co-existing pollutants in the surrounding soil, including antibiotics and metal ions, thereby contributing to composite pollution. However, adsorption does not necessarily imply enhanced toxicity or increased plant uptake. Depending on polymer chemistry, aging status, particle size, pollutant speciation, soil composition, and exposure sequence, microplastics may increase, decrease, or have little effect on the bioavailability of co-existing pollutants. They may act as carriers that facilitate pollutant transport to the rhizosphere, as sinks that reduce the freely available fraction of contaminants, or as additional stressors that indirectly alter pollutant toxicity by modifying soil physicochemical properties and microbial communities. Therefore, the effects of microplastics and co-existing pollutants on terrestrial plants should be interpreted as context-dependent combined effects rather than as uniformly enhanced toxicity. The presence of antibiotics and heavy metals in soil can have negative effects on the soil–plant-microbe system, leading to environmental concerns [111,112]. It has been demonstrated that PE can adsorb ciprofloxacin, whereas the presence of heavy metal ions (Cu^2+^, Cr^3+^, Cr^6+^, Cd^2+^, and Pb^2+^) can affect the adsorption process and contribute to toxic effects [113].

Antibiotics have emerged as important emerging contaminants in the soil environment, and when combined with microplastics, this co-contamination can affect plant health and ecosystem stability. The combination of oxytetracycline (OTC) and PE exposure resulted in a notable decrease in wheat seedling biomass and height. Composite pollution substantially decreased the abundance of certain genera classified as plant growth-promoting rhizobacteria [114]. Similarly, Zhang et al. investigated the impact of PE (0.2%) and norfloxacin/doxycycline (5 mg/kg) on the growth and development of wheat and maize plants. The results indicated that the composition of the bacterial community experienced alterations, including a decline in the abundance of *Kosakonia* and *Sphingomonas*, thereby negatively affecting plant seedling growth [115]. These findings suggest that antibiotic–microplastic co-exposure may affect plants not only through direct phytotoxicity but also through changes in rhizosphere microbial communities. In addition, Li et al. focused on the effects of OTC with PE and PLA on pak choi, resulting in alterations in plant metabolite and amino acid synthesis, disrupting normal plant growth and development [116]. A salient feature of this study is its comparison of the phytotoxic effects of degradable and non-degradable microplastics in combination with antibiotics. The findings demonstrated that the presence of degradable microplastics in conjunction with antibiotics resulted in substantial alterations to soil properties and exerted a more deleterious effect on plant performance than the composite pollution formed by non-degradable microplastics and antibiotics. This comparison indicates that polymer chemistry and degradability can influence the outcome of combined contamination, possibly through changes in soil properties, leachate release, and antibiotic availability.

The effects of co-contamination with microplastics and heavy metals on plants are often different from those of the corresponding single pollutants, impacting plant physiological functions, oxidative stress, nutrient acquisition, and gene expression. Such co-contamination often leads to more complex and severe effects than pollution from individual sources. Dong et al. found that rice plants exposed to heavy metals, arsenic (As), polystyrene microplastics (PSMP), and polytetrafluoroethylene (PTFE) experienced a decrease in phosphorus and organic nitrogen levels, as well as a reduction in the abundance of *Proteobacteria*, leading to delayed plant growth [117]. Co-exposure to PS and As has been shown to trigger the altered expression of key transcription factor genes and impair antioxidant defense systems in rice, thereby affecting plant growth [118]. Also, co-exposure to As and PS has been shown to trigger the differential expression of key transcription factor genes and impair antioxidant defense systems in wheat seedlings (*Triticum aestivum* L.), thereby affecting plant growth [74]. Furthermore, composite pollution involving Cd and microfibres (PMFs) alters metabolite production in lettuce (*Lactuca sativa*) leaves, resulting in significant reductions in the relative abundance of amino acids and sugar alcohols, which are detrimental to plant growth [119]. These studies indicate that heavy metal–microplastic co-exposure can intensify plant stress responses compared with single exposure in some cases.

Conversely, it has been established that composite pollution may also stimulate plant growth. Zong et al. examined the effects of the combined contamination of PS and heavy metals (Cu and Cd) and found that composite pollution has a positive effect on wheat, increasing chlorophyll content, reducing ROS accumulation, and contributing to growth and development [120]. Such contrasting results suggest that microplastics may either exacerbate or mitigate metal-associated phytotoxicity, depending on their influences on metal adsorption–desorption behavior, ion speciation, root available metal fractions, and soil physicochemical conditions. For instance, microplastics with high sorption capacity may reduce the freely available metal fraction and alleviate phytotoxicity, whereas aged or fine particles bearing oxygen-containing functional groups may increase contaminant mobility or promote root-level contaminant exposure. Soil organic matter, clay minerals, soil pH, and dissolved organic carbon can further compete for binding sites or alter metal speciation, thereby modulating the apparent plant response.

Microplastics in soil, along with other pollutants, such as antibiotics and heavy metals, can have adverse effects on plants. These effects could be attributed to microplastics altering the mobility, adsorption, desorption, and plant availability of co-existing pollutants, thereby modifying plant uptake, toxicity, and remediation capacity. Alternatively, this phenomenon could result from positive or negative combined effects between the leachates of other pollutants and the toxic additives present in microplastics [121]. Importantly, combined-exposure effects should be distinguished from statistically verified interactions. In this review, the term “combined effect” refers to scenarios in which co-exposure outcomes differ from single-pollutant exposure or control treatments, whereas “synergistic” or “antagonistic” interactions should be reserved for instances where the observed combined response is statistically compared against an expected additive response, for example, via factorial experimental designs or statistical interaction terms. This distinction is particularly important because a combined treatment that differs from a single-pollutant treatment does not necessarily indicate synergy or antagonism; it may simply reflect additive toxicity, altered exposure, or independent stress responses. Consequently, further research on the mechanisms of synergistic or antagonistic interactions between microplastics and co-existing pollutants in the soil will contribute to understanding the effects of complex pollution on soil properties, soil microorganisms, plant growth, and the transport, accumulation, and removal of pollutants [122]. Future studies should include single-pollutant and combined treatments, quantify contaminant bioavailability, and explicitly test additive versus non-additive responses to better resolve the mechanisms of microplastic-associated composite pollution.

## 4. Uptake, Translocation and Accumulation of Microplastics by Plants

### 4.1. Uptake and Accumulation of Microplastics by Plants

Microplastics in the soil are fragmented by biotic and abiotic processes. The effects of this phenomenon on seedling growth, physiological functions, antioxidant enzyme activities, and gene expression are related to their adsorption on plant surfaces and, in some cases, their possible accumulation within plant tissues. However, current evidence for microplastic uptake and accumulation in plants remains method-dependent and should be assessed in relation to both particle characteristics and experimental design. Several factors may influence the reliability of such conclusions, including particle size, surface charge, polymer type, aging status, labeling method, aggregation behavior, and the experimental matrix. In particular, results obtained using fluorescently labeled particles may be affected by dye leaching, particle aggregation, tissue autofluorescence, and insufficient washing of root surfaces, which can lead to false-positive localization signals. Therefore, studies based only on fluorescence imaging should be distinguished from those that additionally confirm polymer identity within plant tissues using complementary analytical methods.

Plants can accumulate microplastics through the gaps between their lateral roots and epidermal cells [123], and through endocytosis, including vesicle uptake [124]. Additionally, microplastics can adhere to the root surface of plants, subsequently penetrating the epidermis and entering the plant through the cortical tissues [125]. Furthermore, certain submerged aquatic plants can absorb and accumulate microplastics via electrostatic interactions [126]. This process can result in significant accumulation of microplastics in plants. Nevertheless, these observations should be interpreted with care, because particle signals associated with roots may reflect different scenarios, including true internalization, retention within apoplastic spaces, or simple attachment to external root surfaces. The relative contribution of these processes may vary considerably among studies depending on particle properties and analytical approaches.

PS can penetrate cotton roots and is absorbed through intercellular spaces and accumulates in the epidermis, intercellular spaces of cortical tissues, and root xylem ducts [107]. Taylor et al. demonstrated that 1 µm-sized PS can accumulate in the root crown cells of wheat, while it was not clearly observed in deeper root tissues [127]. Furthermore, Erdem et al. found that the principal aggregation sites of PP in maize and wheat are distinct, with most microplastics in the stems of maize and the roots of wheat, and a smaller quantity present in the leaves [128]. As demonstrated by the preceding studies, the presence of various plant species has been shown to exert a significant influence on the processes of microplastic uptake and subsequent accumulation. Research has suggested that the size of microplastic particles plays a significant role in their uptake and accumulation in plants, with smaller particles exhibiting a greater tendency to accumulate [129]. For example, the experiment involved the exposure of maize (*Zea mays*) seedlings to small PS beads (0.2, 0.5, and 1.0 μm) and large PS beads (2.0 and 5.0 μm) for a period of seven days. The results indicated that the small PS beads significantly increased the accumulation and distribution of PS beads in the roots [130]. This pattern is broadly consistent with the view that smaller particles have greater mobility and are more likely to interact with plant tissues. However, particle size alone is unlikely to determine plant uptake, because surface charge, functional groups, polymer composition, aging-induced surface changes, and aggregation in different exposure media may also strongly influence particle behavior at the root–plant interface.

Plants exhibit a range of distinctive structural characteristics including root systems, xylem, and vesicles. In addition, they possess specific physiological traits, such as transpiration. These attributes promote the absorption and deposition of microplastics in various tissues and plants [131]. Different plants absorb and accumulate microplastics, with the root system being the primary site of accumulation. One possible explanation for this phenomenon is that the plant root system is not a closed system and the cellular gaps between the epidermal cells form an open space that facilitates the entry of microplastics into the plant root system. In addition, the apical epidermal cells of the root system become deformed as a result of microplastics, enlarging the cellular gaps and causing deformations and fractures, which in turn lead to the swelling and deformation of the mature root zone, facilitating the entry and accumulation of microplastics within the plant root system [132,133]. At the same time, these interpretations should not be generalized to all studies or all particle classes. In particular, for larger microplastics, observations of particle association with root tissues do not necessarily demonstrate passage across intact biological barriers. In some cases, the reported accumulation may partly reflect strong adsorption to root surfaces or trapping within damaged or loosely structured outer tissues rather than unequivocal internal uptake.

Microplastics have been reported to negatively affect plant growth and development by interfering with water and nutrient uptake, thereby potentially increasing ecological and food-safety risks [76]. However, the mechanisms underlying these effects, particularly the relative contributions of surface adsorption, apoplastic retention, and true internalization, remain to be fully clarified. Therefore, further research is needed to better understand the distribution, translocation, and accumulation of microplastics in terrestrial plants. Future studies should place greater emphasis on methodological rigor and standardization, including thorough surface-cleaning protocols, appropriate negative controls, and the combined use of imaging and polymer-specific analytical techniques, so that the presence and identity of microplastics within plant tissues can be more reliably confirmed. In addition, particle size, surface charge, polymer type, aging status, aggregation behavior, and the complexity of the experimental matrix should be systematically considered in order to improve the interpretation of plant uptake and accumulation.

### 4.2. Translocation of Microplastics by Plants

The roots and leaves of plants represent the primary entry points for microplastics, whereas cell walls serve as the primary barrier for microplastics to their penetration into plant tissues. Microplastics have been reported to induce the deformation of plant cell walls, thereby creating larger pores through which larger microplastics can gain access to plants [134]. However, the extent to which such structural changes facilitate the entry of different microplastic particles may vary depending on particle properties, plant species, and exposure conditions. It has been demonstrated that microplastics can enter and accumulate in plants and migrate to other organs and tissues within the plant via intercellular channels and extracellular transport networks [135]. Nevertheless, evidence for microplastic translocation should be interpreted in relation to the analytical methods used, because particle detection in vascular-associated regions does not necessarily confirm long-distance internal transport. Given the small pore-size exclusion limit of plant cell walls and the barrier function of the endodermis and Casparian strip, the passage of intact micron-sized microplastics across these structures is unlikely under normal physiological conditions. Therefore, reported crack-entry or apoplastic movement should be interpreted as potential access to outer root tissues, sites of barrier discontinuity, or damaged tissues, rather than as definitive evidence of crossing intact endodermal barriers.

Plant structures are interconnected, forming complex and integrated systems. For instance, the stems and roots are connected through different levels of cell differentiation to form the axes of the plant body, which serve as channels for transporting substances. Furthermore, the xylem serves as the plant transport tissue, facilitating the movement of water and nutrients to various organs. These structures ensure the proper functioning of various physiological functions and may also be a significant factor in the movement of microplastics within the plant body.

**Figure 3:**
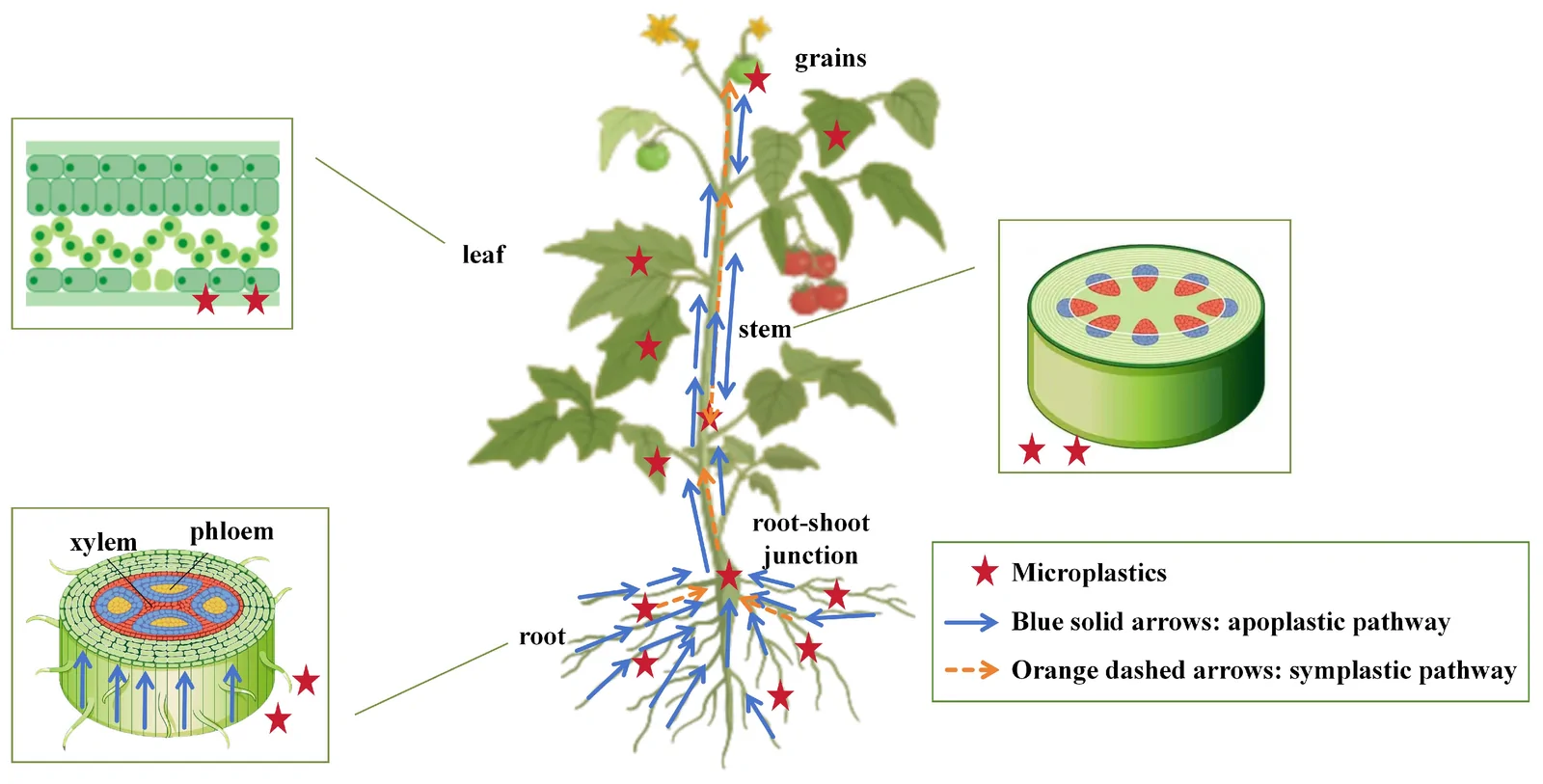
Proposed transport routes of microplastics in plants. Microplastics may associate with or enter root tissues and may subsequently be transported to the root-shoot junction, stem, leaves, and grains through apoplastic or symplastic pathways under certain conditions. Blue solid arrows indicate the apoplastic pathway, orange dashed arrows indicate the symplastic pathway, and red stars represent microplastics.

Studies have demonstrated that once microplastics enter the plant root system, they may be transported to plant stems and leaves via multiple processes, including root pressure and transpiration [136]. The water potential gradient resulting from plant transpiration has been identified as an important driver of microplastic migration in plant vascular systems. For instance, Li et al. discovered that PS was built up in the xylem cell walls and conduits of wheat roots and subsequently migrated to the stems via the vascular system [96]. In addition, sweet potato seedlings were incubated in soil containing PE for a period of three days. It was observed that PE was absorbed by root epidermal cells and gradually migrated inward into the cortex and upward through the mid-column into the epidermal and endodermal tissues of the stem [137]. The migration of 26 μm PE from soil to root of the maize, root to stem, and stem to leaf was reported as 1.07%, 0.76%, and 103.28%, respectively, thereby further supporting the phenomenon of microplastic migration in plants [138]. However, direct comparisons among studies remain difficult because translocation efficiency may be influenced by particle size, polymer type, surface properties, plant species, and exposure matrix. Furthermore, Zhao et al. exposed water spinach (*Ipomoea aquatica* F.) to 1 µm PS particles, demonstrating that PS was absorbed by the roots and then moved to the leaves of the plant [139]. The roots of lettuce subjected to PS (0.2 µm,1.0 µm) for 14 days absorbed significant quantities of PS, which subsequently migrated to the above-ground parts of the plant and accumulated in large amounts in the directly edible stems and leaves (Figure 3) [140].

In addition to the processes of distribution, accumulation, and transport within plants, microplastics can migrate to other vectors or organisms, including insects and humans. Given that atmospheric deposition is a significant source of microplastics in soil, it is probable that these particles will be captured by different plants through atmospheric deposition and subsequently attached to the aboveground parts of plants, such as leaves [141]. Moreover, it is possible that microplastics that find their way into edible plant portions will move up the food chain to higher trophic levels [142,143]. The transformation and accumulation of microplastics at higher trophic levels can have several negative effects, most of which are detrimental to ecosystems and human health [133]. However, the mechanisms underlying microplastic translocation within plants and their subsequent transfer across trophic levels remain unclear. Elucidating these migration mechanisms in future studies will provide a theoretical foundation for maintaining ecological stability.

## 5. Conclusions and Outlook

### 5.1. Conclusions

Microplastics are emerging pollutants that have attracted increasing attention in recent years. This review summarizes the current knowledge on microplastics, with particular emphasis on their sources and distribution in soils and their effects on the soil–plant system. A growing body of evidence indicates that microplastics can enter the soil system and accumulate in the soil, thereby altering soil physicochemical properties, including pH, soil organic matter, electrical conductivity, water-holding capacity, and enzyme activities. They can also influence the diversity and structure of rhizosphere microbial communities, which may further affect plant growth and development.

In addition, microplastics may adsorb to root surfaces and, under certain conditions, enter plant tissues through root tips or other vulnerable sites, although fluorescence-based evidence for such processes remains subject to methodological uncertainty. Once associated with or taken up by plants, they may accumulate in roots and, in some cases, be translocated to aboveground tissues, thereby inducing oxidative stress and affecting seed germination and seedling development. These effects may subsequently influence germination rate, plant height, biomass, photosynthesis, and antioxidant enzyme activities. Furthermore, microplastics may exert cytotoxic and genotoxic effects, thereby influencing plant metabolism and nutrient uptake. The coexistence of microplastics with toxic substances or remediation materials may further modify their effects on plant growth, with potential consequences for ecosystem stability and human health.

### 5.2. Outlook

#### 5.2.1. Detection of Microplastics

Humans use large quantities of plastic products in their everyday lives, with millions of tons of plastic released into the ecosystem each year [5]. Microplastics are generated when plastics break down and are released into the environment, and they remain challenging to detect. Currently, a limited number of methods are available for the qualitative or quantitative analysis of microplastics, as well as for the determination of their level of harm and for evaluating degradation [144]. Nile red staining and fluorescence-based techniques have been employed to detect microplastics in aquatic ecosystems, sewage sludge, and street dust [24,145]. However, extending these approaches to soil–plant systems remains challenging, because fluorescence signals may be influenced by dye leaching, particle aggregation, tissue autofluorescence, and incomplete removal of surface-bound particles [146]. Currently, microscopy (scanning electron, fluorescence, optical, and stereo microscopy), spectroscopy (Pyr-GC-MS, LC-MS/MS, and FT-IR), and other techniques are currently used to identify and measure isolated and pretreated microplastics [147]. Because the detection and quantification of microplastics depend on particle type, abundance, and environmental matrix, a single analytical method is unlikely to be sufficient. Therefore, future efforts should prioritize the development of standardized and integrated methodologies for the isolation, identification, and quantification of microplastics in soil and plant systems, enabling more reliable qualitative and quantitative assessments [148].

#### 5.2.2. Effects of Microplastics on Metal-Hyperaccumulating Plants and Implications for Phytoremediation

Microplastics in soil frequently coexist with other contaminants, particularly heavy metals and metalloids, and such co-contamination may alter rhizosphere processes, pollutant bioavailability, and plant responses. In this context, metal-hyperaccumulating plants deserve particular attention because they are widely studied for the phytoremediation of contaminated soils. However, most available studies have focused on common crop species under microplastic-only exposure, whereas the effects of microplastics on metal-hyperaccumulating plants remain insufficiently understood. Moreover, when evaluating the joint effects of microplastics and co-occurring contaminants, genuine interaction effects should be distinguished from simple comparisons between single and combined exposure treatments, and robust conclusions should preferably be supported by evidence of non-additive biological responses.

Current evidence suggests that microplastics may influence the growth and physiological performance of metal-hyperaccumulating plants, either directly or indirectly through changes in soil properties and microbial communities. For example, the addition of microplastics has been reported to alter heavy metal availability in soil [117] and modify the diversity of soil microbial communities [114], which may subsequently affect plant growth and contaminant uptake. In *Solanum nigrum* L., increasing concentrations of PE reduced root length and leaf area, indicating that microplastics may inhibit the growth of this metal-hyperaccumulating species [149]. These findings suggest that microplastics may interfere with the ecological function and remediation performance of hyperaccumulating plants under contaminated soil conditions.

Metal-hyperaccumulating plants are characterized by their ability to absorb and accumulate specific heavy metals or metalloids from contaminated soils, which underlies their potential application in phytoremediation. For example, *Lantana camara* and *Solanum photeinocarpum* have been used as Cd-accumulating species to investigate how different types of microplastics and heavy metals jointly affect plant growth and remediation-related processes. Previous work has shown that microplastics can negatively affect plant growth and alter soil biochemical properties relevant to heavy metal remediation, as reflected by significant decreases in soil pH, dehydrogenase activity, and phosphatase activity [150]. These findings suggest that microplastics may modify the soil–plant environment in ways that influence heavy metal phytoremediation. Nevertheless, because the hyperaccumulation concept was originally defined for specific metallic elements, whether and to what extent it is applicable to microplastic uptake, retention, and removal within plant–soil systems remains to be elucidated.

Accordingly, observations of microplastic particles on root surfaces or within root tissues are more appropriately regarded as evidence of particle attachment and retention at the plant–soil interface, rather than direct proof of effective phytoremediation capacity. To evaluate the potential remediation capacity of hyperaccumulating plants for microplastic-polluted soils, future studies should adopt a comprehensive and systematic evaluation framework including system-level removal efficiency, accurate in situ identification of microplastics in plant tissues, root-to-shoot translocation capacity, microplastic accumulation in harvestable plant biomass, and quantitative mass-balance analysis before and after phytoremediation. Furthermore, the environmental implications of these phytoremediation processes should be assessed from a broader perspective, particularly regarding the safe disposal of microplastic-contaminated biomass and the potential trophic transfer of retained microplastics to terrestrial food webs.

Taken together, current studies suggest that microplastics can affect the growth, physiological status, and remediation-related performance of metal-hyperaccumulating plants, particularly under conditions of co-contamination with heavy metals. Nevertheless, whether these plants can make a meaningful contribution to the simultaneous mitigation of heavy metals and microplastics in contaminated soils remains largely unexplored. Addressing this issue will require further investigation into the effects of microplastics on metal uptake, plant physiological responses, and rhizosphere processes across different hyperaccumulating species, together with system-level assessments of their potential role in the remediation of microplastic-contaminated soils.

#### 5.2.3. Combined Effects of Microplastics and Soil Remediation Materials on Plants

Biochar and other soil amendment materials are widely used to improve soil quality, support plant growth, and, in some cases, reduce the bioavailability of soil contaminants. However, microplastic pollution in soil ecosystems has been demonstrated to adversely affect plant growth and development. Nevertheless, incorporating biochar or specific soil amendments has been shown to partially mitigate these adverse effects.

Biochar has been widely investigated as a soil amendment and remediation material and is generally considered to exert fewer adverse effects on soil biota and plants than many conventional chemical amendments. Several studies have demonstrated that biochar can mitigate the negative effects of microplastics on plants. For instance, Elbasiouny et al. investigated the effects of varying concentrations of microplastics (acrylic plastic, 1.5%, 7.5%, and 15%) on soil characteristics and the growth of the broad bean (*Vicia faba*). The addition of microplastics resulted in decreases in chlorophyll content and plant biomass. However, the incorporation of biochar (BC: 2%) mitigated or reversed these adverse effects [151]. It is reported that the adverse effects of PE on barley (*Hordeum vulgare*) were alleviated by incorporating biochar, resulting in increased water content and chlorophyll levels [33]. In addition, PVC alone significantly inhibits lettuce growth and causes oxidative damage. However, the addition of corncob-generated biochar altered the negative effect of PVC on lettuce growth [152]. Together, these studies suggest that biochar may alleviate some of the adverse effects of microplastics on plants [153].

In addition to biochar, other emerging soil amendments may also modify plant responses in microplastic-contaminated soils. For instance, dissolved organic matter and hydrocarbon-rich compounds (HBC) have been utilized as soil conditioners. A study has shown the effect of the coexistence of microplastics with HBC substances on the growth of wheat. The results suggest that this interaction enhances plant urease activity and facilitates nitrogen uptake, thereby promoting positive outcomes in terms of wheat growth [40]. The novel soil amendment known as SynCom was the focus of this investigation. Research indicated that SynCom reduced the inhibitory effects of peanut growth caused by tyre-derived rubber crumb (RC), a microplastic, thereby enhancing peanut biomass and antioxidant enzyme activity [154].

Employing biochar and soil amendments has been demonstrated to mitigate the effects of microplastics on plants, and this amelioration occurs via multiple mechanisms. One mechanism underlying the beneficial effects of biochar under microplastic stress may be the enhancement of soil pH buffering capacity following biochar amendment [155]. This addition also increases soil organic matter and alters its composition [156]. Furthermore, the detrimental effects of microplastics on the soil–plant system can be mitigated by biochar addition. This is likely because biochar and other soil conditioners can serve as remediation agents and possess several advantageous properties, including strong adsorption effects and high selectivity [157]. Biochar and other soil conditioners hold promise as effective agents for pollutant removal. However, the effectiveness of these materials is likely to depend on amendment type, microplastic properties, soil conditions, and plant species. Further studies are therefore needed to clarify the mechanisms underlying these interactions and to evaluate their long-term significance in soil–plant systems.

#### 5.2.4. Control of Microplastics

Reducing microplastic risks in soil–plant systems requires more than simply decreasing visible plastic residues; it also requires limiting their persistence, bioavailability, and ecological impacts within the soil–plant-rhizosphere continuum. In this context, control strategies should include source reduction, substitution with less harmful materials, interception or immobilization in soil, and degradation pathways that truly reduce environmental persistence rather than merely transform larger particles into smaller ones.

From a mechanistic perspective, it is important to distinguish between fragmentation, depolymerization, biodegradation, and complete mineralization when evaluating microplastic control technologies. Fragmentation only reduces particle size and may even increase particle mobility and bioavailability in soil. Depolymerization refers to the cleavage of polymer chains into smaller molecular units, which does not necessarily imply biological assimilation or detoxification. Biodegradation generally involves the microbial or enzymatic conversion of polymers into lower-molecular-weight compounds, whereas complete mineralization represents the ultimate conversion of polymer carbon into inorganic end-products such as CO_2_, H_2_O, and, under certain conditions, CH_4_. For soil remediation purposes, these processes should not be used interchangeably, because a treatment that induces fragmentation without substantial mineralization may fail to reduce, and may even aggravate, ecological risks.

Current approaches for microplastic mitigation include biological degradation, catalytic or photocatalytic degradation, and the development of alternative materials [158]. Biodegradation may be mediated by enzymes and microorganisms, including bacteria, fungi, and microbial consortia [159]. However, its efficiency in soil is often constrained by polymer type, crystallinity, additive composition, particle size, aging status, and environmental conditions such as moisture, temperature, oxygen availability, and nutrient supply. Moreover, evidence of surface weathering or partial mass loss should not be equated with complete degradation. Photocatalytic processes can also promote polymer breakdown through direct or indirect oxidative pathways [160], but their applicability in soil–plant systems remains limited by light penetration, catalyst stability, soil heterogeneity, and the uncertain fate of intermediate products. Therefore, the practical significance of these methods for rhizosphere remediation requires more rigorous validation under realistic soil conditions.

In addition to degradation-based strategies, the replacement of conventional plastics with alternative materials has received increasing attention [161]. However, such substitution should be evaluated critically rather than assumed to be inherently environmentally benign. Although cellulose-based materials derived from agricultural biomass are often considered promising because of their biodegradability, low toxicity, and renewable origin [162], not all alternative plastics or biodegradable polymers behave similarly in soil. As discussed above, some biodegradable plastics, such as PLA and PBAT, may under certain conditions exert comparable or even stronger effects on soil physicochemical properties and microbial communities than conventional plastics. Their promotion as eco-friendly alternatives should therefore be based on demonstrated environmental performance in relevant soil systems, including degradation behavior, additive composition, by-product formation, and ecological consequences for plants and rhizosphere biota.

Overall, effective control of microplastics in terrestrial ecosystems should not be defined simply by visible particle reduction, but by whether a given strategy lowers microplastic persistence, bioavailability, and ecological risk in soil–plant systems. Future work should therefore integrate standardized analytical methods, mechanistic differentiation among transformation pathways, and realistic assessments of plant, rhizosphere, and soil ecological responses, so as to determine whether proposed control measures deliver meaningful environmental benefits under field-relevant conditions.

## Data Availability

No new data were created or analyzed in this study. Data sharing is not applicable to this article.

## Supplementary Materials

The following supporting information can be downloaded at: https://www.mdpi.com/article/10.3390/molecules31173028/s1, Table S1. Representative studies on the sources and distribution characteristics of microplastics in soils. References [21,163,164,165,166,167,168,169,170,171] are cited in the Supplementary Materials.
