# Supplementary material for: Interaction of Microplastics, Plants, and Rhizosphere: A Critical Review
Source: Molecules. 2026 Aug 28;31(17):3028. doi: 10.3390/molecules31173028 (PMC13567708; doi:10.3390/molecules31173028)
Supplement: Supplementary file 1 [file molecules-31-03028-s001.zip › molecules-4501708-supplementary.pdf]

Supplementary Table S1. Representative studies on the sources and distribution characteristics of microplastics in soils.

Source category	Source description	Study region	Environmental matrix	Polymer types	Particle size range	Reported abundance	Reference
Agricultural inputs	Residual plastic mulch contamination	Baoji, China	Agricultural soil	Not reported	<1 mm, 1-2 mm, 2-5 mm	1,974-3,656 items/kg	[1]
Agricultural inputs	Mulch residues	Hangzhou Bay, China	Agricultural soil	PE, PP	1-3 mm	571 items/kg	[2]
Agricultural inputs	Residual plastic mulch degradation	Xinjiang and 19 provinces, China	Agricultural soil	PE	<5 mm	80.3±49.3-1,075.6±346.8 items/kg	[3]
Agricultural inputs	Mulch film-derived microplastic inputs	Franconia, Germany	Agricultural soil	PE, PS, PP	1-5 mm	0.34±0.36 particles/kg	[4]
Organic amendments	Sewage sludge application	Southwestern Poland	Sludge-amended agricultural soil	Plastic fibers	Not reported	4,050±2,831 particles/kg	[5]

Organic amendments	Sludge-associated microplastic inputs	Mauritius	Agricultural soil	PP	<0.5 mm	14,750±8,612.9 particles/kg	[6]
Organic amendments	Compost-associated microplastic inputs	Southern England	Agricultural soil	PA, PE, PET, PP, PS, PVA	Not reported	1,420 and 1,480 MPs/kg (dw)	[7]
Organic amendments	Sewage sludge application	Mellipilla, central Chile	Agricultural soil	Plastic fibers	0.97 mm	1.1-3.5 particles/g	[8]
Mixed anthropogenic inputs	Textile fiber-derived and other anthropogenic inputs	Shaanxi, China	Agricultural soil	PS, PE, PP, HDPE, PVC	0-0.49 mm	1,430-3,410 items/kg	[9]
Mixed anthropogenic inputs	Combined agricultural and peri-urban inputs	Shanghai, China	Peri-urban agricultural soil	PP, PE	0.03-5 mm	62.5 items/kg	[10]

Note: The data presented under polymer types, particle size range, and reported abundance were retained as reported in the original studies. The studies were grouped according to the primary source category emphasized in the original articles.

Reference

[1] Song, D.; Ma, L.; Wang, Q. Occurrence characteristics and environmental effects of microplastics in typical farmland soils in Baoji area. J. Arid Land Res. Environ. 2021, 35, 170-175.

[2] Zhou, B.; Wang, J.; Zhang, H.; Shi, H.; Fei, Y.; Huang, S.; Tong, Y.; Wen, D.; Luo, Y.; Barceló, D. Microplastics in agricultural soils on the coastal plain of Hangzhou Bay, east China: Multiple sources other than plastic mulching film. J. Hazard. Mater. 2020, 388, 121814. https://doi.org/10.1016/j.jhazmat.2019.121814

[3] Huang, Y.; Liu, Q.; Jia, W.; Yan, C.; Wang, J. Agricultural plastic mulching as a source of microplastics in the terrestrial environment. Environ. Pollut. 2020, 260, 114096. https://doi.org/10.1016/j.envpol.2020.114096

[4] Piehl, S.; Leibner, A.; Löder, M. G. J.; Dris, R.; Bogner, C.; Laforsch, C. Identification and quantification of macro- and microplastics on an agricultural farmland. Sci. Rep. 2018, 8, 1. https://doi.org/10.1038/s41598-018-36172-y

[5] Medyńska-Juraszek, A.; Szczepańska, A. Microplastic Pollution in EU Farmland Soils: Preliminary Findings from Agricultural Soils (Southwestern Poland). Agriculture. 2023, 13, 1733. https://doi.org/10.3390/agriculture13091733

[6] Ragoobur, D.; Huerta-Lwanga, E.; Somaroo, G. D. Microplastics in agricultural soils, wastewater effluents and sewage sludge in Mauritius. Sci. Total Environ. 2021, 798, 149326. https://doi.org/10.1016/j.scitotenv.2021.149326

[7] Zapata, R.; Douglas, P.; Chan, K. L. A.; Barratt, B.; Wright, S. L. Microplastic emissions via air and compost from an industrial composting facility in England. Environ. Pollut. Bioavailability. 2023, 36, 1. https://doi.org/10.1080/26395940.2023.2296046

[8] Corradini, F.; Meza, P.; Eguiluz, R.; Casado, F.; Huerta-Lwanga, E.; Geissen, V. Evidence of microplastic accumulation in agricultural soils from sewage sludge disposal. Sci. Total Environ. 2019, 671, 411-420. https://doi.org/10.1016/j.scitotenv.2019.03.368

[9] Ding, L.; Zhang, S.; Wang, X.; Yang, X.; Zhang, C.; Qi, Y.; Guo, X. The occurrence and distribution characteristics of microplastics in the agricultural soils of Shaanxi Province, in north-western China. Sci. Total Environ. 2020, 720, 137525. https://doi.org/10.1016/j.scitotenv.2020.137525

[10] Liu, M.; Lu, S.; Song, Y.; Lei, L.; Hu, J.; Lv, W.; Zhou, W.; Cao, C.; Shi, H.; Yang, X.; He, D. Microplastic and mesoplastic pollution in farmland soils in suburbs of Shanghai, China. Environ. Pollut. 2018, 242, 855-862. https://doi.org/10.1016/j.envpol.2018.07.051
